# A Porcine Sepsis Model With Numerical Scoring for Early Prediction of Severity

**DOI:** 10.3389/fmed.2022.867796

**Published:** 2022-05-09

**Authors:** Attila Rutai, Bettina Zsikai, Szabolcs Péter Tallósy, Dániel Érces, Lajos Bizánc, László Juhász, Marietta Zita Poles, József Sóki, Zain Baaity, Roland Fejes, Gabriella Varga, Imre Földesi, Katalin Burián, Andrea Szabó, Mihály Boros, József Kaszaki

**Affiliations:** ^1^Institute of Surgical Research, Albert Szent-Györgyi Medical School, University of Szeged, Szeged, Hungary; ^2^Institute of Medical Microbiology, Albert Szent-Györgyi Health Center and Medical School, University of Szeged, Szeged, Hungary; ^3^Department of Laboratory Medicine, Albert Szent-Györgyi Health Center, University of Szeged, Szeged, Hungary

**Keywords:** sepsis, septic shock, fecal peritonitis, organ dysfunction, SOFA score, pig model, inflammatory markers

## Abstract

**Introduction:**

Sepsis can lead to organ dysfunctions with disturbed oxygen dynamics and life-threatening consequences. Since the results of organ-protective treatments cannot always be transferred from laboratory models into human therapies, increasing the translational potential of preclinical settings is an important goal. Our aim was to develop a standardized research protocol, where the progression of sepsis-related events can be characterized reproducibly in model experiments within clinically-relevant time frames.

**Methods:**

Peritonitis was induced in anesthetized minipigs injected intraperitoneally with autofeces inoculum (*n* = 27) or with saline (sham operation; *n* = 9). The microbial colony-forming units (CFUs) in the inoculum were retrospectively determined. After awakening, clinically relevant supportive therapies were conducted. Nineteen inoculated animals developed sepsis without a fulminant reaction. Sixteen hours later, these animals were re-anesthetized for invasive monitoring. Blood samples were taken to detect plasma TNF-α, IL-10, big endothelin (bET), high mobility group box protein1 (HMGB1) levels and blood gases, and sublingual microcirculatory measurements were conducted. Hemodynamic, respiratory, coagulation, liver and kidney dysfunctions were detected to characterize the septic status with a pig-specific Sequential Organ Failure Assessment (pSOFA) score and its simplified version (respiratory, cardiovascular and renal failure) between 16 and 24 h of the experiments.

**Results:**

Despite the standardized sepsis induction, the animals could be clustered into two distinct levels of severity: a sepsis (*n* = 10; median pSOFA score = 2) and a septic shock (*n* = 9; median pSOFA score = 8) subgroup at 18 h of the experiments, when the decreased systemic vascular resistance, increased DO_2_ and VO_2_, and markedly increased ExO_2_ demonstrated a compensated hyperdynamic state. Septic animals showed severity-dependent scores for organ failure with reduced microcirculation despite the adequate oxygen dynamics. Sepsis severity characterized later with pSOFA scores was in correlation with the germ count in the induction inoculum (*r* = 0.664) and CFUs in hemocultures (*r* = 0.876). Early changes in plasma levels of TNF-α, bET and HMGB1 were all related to the late-onset organ dysfunctions characterized by pSOFA scores.

**Conclusions:**

This microbiologically-monitored, large animal model of intraabdominal sepsis is suitable for clinically-relevant investigations. The methodology combines the advantages of conscious and anesthetized studies, and mimics human sepsis and septic shock closely with the possibility of numerical quantification of host responses.

## Introduction

Sepsis is defined as life-threatening organ dysfunction caused by a dysregulated host response to infection (1). Clinical characteristics have repeatedly been standardized in recent decades, and today the Sequential (sepsis-related) Organ Failure Assessment (SOFA) scoring system adequately characterizes the level of dysfunction of vital organs (2). In parallel, the recently established MQTiPSS (Minimum Quality Threshold in Pre-Clinical Sepsis Studies) recommendations for study designs enables us to standardize experimental sepsis, including the assessment of organ failure/dysfunction parameters (as described in Recommendation 12), which reflect the specificities of the human disease (3).

Nevertheless, it is recognized that the development of septic signs is determined by a number of individual reactions, all of which can mask the effects of interventions (4). Porcine models of sepsis have many advantages over rodent studies because domestic pigs are more closely related to humans in terms of anatomy, genetics and physiology (5, 6). Furthermore, pigs are more suitable for clinically-relevant anesthesia, instrumentation and intensive care, including invasive hemodynamic monitoring, fluid resuscitation and repetitive blood sampling (7). These characteristics allow for a more standardized induction and individual evaluation of disease progression and severity (8–10), but the need for anesthesia and observation time are still restrictive or limiting factors (8–10).

Unlike in many other areas, basic research with laboratory animals has not resulted in major therapeutic breakthroughs in this field, with one of the possible reasons being that better models are needed to translate the experimental results into clinical practice (4, 7, 11).

Based on this background, our objective was to improve the design of swine models of sepsis, with the final goal being to reduce heterogeneity and the gap between the messages of animal and human studies. In this context, our aim was to establish a standardized protocol, where the progression of events can be characterized in sufficient detail by a pig-specific SOFA (pSOFA) scoring system without the confounding effects of continuous anesthesia (10, 12).

In this line, tracking changes in oxygen dynamics and plasma mediators may be crucial in assessing the severity of the evolving septic reaction and organ damage (13–16). We hypothesized that the dynamics of typical indicators of systemic inflammation, such as tumor necrosis factor alpha (TNF-α) and interleukin-10 (IL-10), or biomarkers of tissue hypoxia and necrosis, such as big endothelin (bET) and high-mobility group box 1 protein (HMGB1), may be associated with numerical pSOFA score changes and that this relationship can therefore be useful in recognizing the development of organ dysfunctions in experimental sepsis.

## Materials and Methods

### Animals

The experiments were performed on outbred Vietnamese minipigs of both sexes (*n* = 36, 35 ± 9 kg bw) in accordance with National Institutes of Health guidelines on the handling of and care for experimental animals and EU Directive 2010/63 on the protection of animals used for scientific purposes (approval number V/175/2018). The animals were fasted for 12 h with free access to tap water before the start of the procedures.

### Experimental Protocol

The study was divided into four stages: (1) baseline measurements and baseline blood sampling under temporary anesthesia; (2) induction of sepsis with intraperitoneal (ip) fecal inoculum or sham-operation with ip sterile saline at time zero of the experiment; (3) progression of sepsis in extubated, unrestrained, awake animals for 15 h; and (4) re-anesthesia, instrumentation and monitoring between 16 and 24 h with pSOFA scoring. The experimental protocol for the sham-operated animals followed the same four stages.

Sample size was estimated with a 0.33 ratio of control to experimental animals assuming ~20% mortality after septic induction. If the presumed true hazard ratio of septic subjects relative to controls is 0.2 with a power of 1 – β = 0.9 and the Type I error probability is α = 0.05, the inclusion of nine control and 27 septic animals was recommended based on the survival estimate.

Animals were randomly assigned into sham-operated (Group 1, *n* = 9) and septic (Group 2, *n* = 27) groups. As no septic reaction was observed in five animals in the septic group during the entire study period, these non-responders were later excluded from the study. A further three animals had to be humanely euthanized before stage 4 due to the fulminant septic reaction they developed (see below). Therefore, data from *n* = 19 animals in the septic group were finally used in the subsequent evaluations.

**Figure 1:**
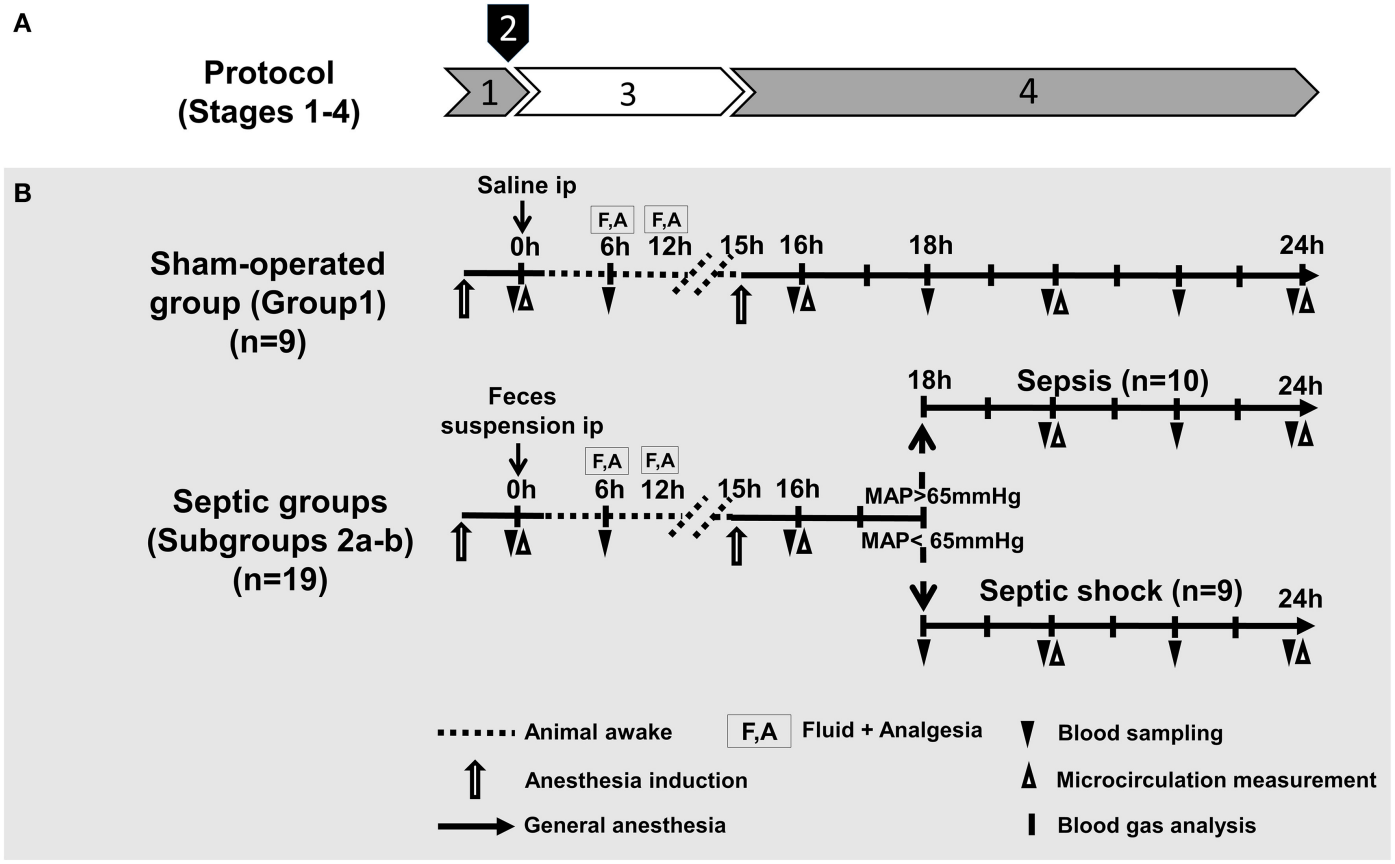
Experimental stages **(A)**, experimental protocol, groups, interventions and assessments **(B)**. **(A)** Experimental stages: (1) baseline measurements (under temporary anesthesia); (2) sepsis induction with intraperitoneal (ip) fecal inoculum; (3) sepsis progression (in awake animals); and (4) re-anesthesia, instrumentation, monitoring and scoring **(B)**. Groups: The animals were randomly assigned into sham-operated and septic groups. Based on disease severity and vasopressor requirement (at 18 h after sepsis induction), animals in the septic group were allocated into sepsis and septic shock subgroups.

Based on disease severity and vasopressor requirement, septic animals were allocated into two subgroups, a sepsis (Subgroup 2a; *n* = 10) and a septic shock group (Subgroup 2b; *n* = 9) 18 h after induction. More specifically, the allocation was based on the consensus criteria of the ‘Sepsis-3' definitions (sepsis involves proven organ damage, with a SOFA score ≥2; lactate level ≥2 mmol L^−1^; septic shock is sepsis with persistent hypotension, requiring vasopressors to maintain mean arterial pressure (MAP) ≥65 mmHg despite adequate fluid resuscitation) (Figure 1).

#### Stage 1. Baseline Measurements and Baseline Blood Sampling Under Temporary Anesthesia

Anesthesia was induced with an intramuscular mixture of tiletamine-zolepam (Zoletil, Virbac, Carros, France 5 mg kg^−1^) and xylasin (2 mg kg^−1^) and continued with an infusion of a mixture of propofol (6 mg kg^−1^h^−1^ iv; Fresenius Kabi, Bad Homburg, Germany), fentanyl (0.02 mg kg^−1^h^−1^; Richter Gedeon, Budapest, Hungary) and midazolam (1.2 mg kg^−1^ h^−1^; Torrex Chiesi Pharma, Vienna, Austria) through an ear vein Braun cannula. A permanent central venous catheter with three lumina (7 F; Edwards Lifesciences LLC, Irvine, U.S.A) was introduced into the jugular vein using an aseptic surgical technique for fluid therapy and blood sampling.

Endotracheal intubation was performed, and the animals were ventilated mechanically (Carescape R860, GE Healthcare, Madison, Wisconsin, U.S.A.). Basic ventilation settings [respiratory rate (RR): 10–12 min^−1^; tidal volume (TV): 8 mL kg^−1^; positive end-expiratory pressure (PEEP): 4–5 cmH_2_O; fraction of inspired oxygen (FiO_2_): 21%] were checked by pulse oximetry (OxiPen^®^ EnviteC, Wismar, Germany) using a sensor fitted to the tongue of the animals. The adequacy of the depth of anesthesia was assessed by monitoring the jaw tone and the pain reaction of the hind leg regularly. The central venous catheter was used for blood sampling at *t* = 0 h and for fluid administration (10 mL kg^−1^ Ringerfundin^®^, B. Braun, Melsungen, Germany). Heart rate (HR) and oxygen saturation were detected by pulse oximetry, and the sublingual microcirculation was monitored by intravital videomicroscopy (see below).

#### Stage 2. Sepsis Induction

Polymicrobial peritonitis was induced with an ip administered autologous feces suspension. Fecal induction inoculum was injected through a 1-cm incision at the umbilicus using a blunt-pointed 12G Veress needle at *t* = 0 h. The sham-operated animals received 200 mL sterile saline ip in the same manner.

#### Preparation of the Sepsis-Inducing Fecal Inoculum

Autologous fresh feces was collected and suspended in 200 ml saline with a 0.6 g kg^−1^ final concentration 6 h before the scheduled intra-abdominal injection. The inoculum was incubated in a water bath for 6 h at 37°C, and the suspension was filtered to remove the pellet. For microbiological analysis, 0.1 mL samples were taken from the suspension before ip administration to determine the microbial concentration (in colony-forming units; CFU mL^−1^) with the standard viable plate count method under aseptic conditions. Readings of CFUs were obtained retrospectively 12 h after sepsis induction and converted to the cell numbers per milliliter inoculum.

The microbial composition of the inoculum was analyzed for the most frequent species by MALDI-TOF mass spectrometry (MS; Bruker Daltonics, Germany) as described earlier (17). Briefly, parallel to the injection of the sepsis inoculum to the animals, 0.1 mL of the fecal suspension was spread on Mueller–Hinton solid media (Bio-Rad, Budapest, Hungary) to isolate the aerobic strains after a 12-h incubation period (37°C). Anaerobic strains were inoculated on a Columbia agar base (Oxoid, Budapest, Hungary) supplemented with 5% (v/v) bovine blood, hemin (1 mg/mL) and vitamin K1 (5 mg/mL) for 48 h (37°C). Fungal and yeast species were isolated on Sabouraud dextrose agar (Bio-Rad, Budapest, Hungary). The spectra from the microbiological samples were acquired using the Microflex LT system (Bruker Daltonik, Bremen, Germany) and analyzed with MALDI Biotyper 3.3 (Bruker, Daltonik) software (18).

#### Stage 3. Sepsis Progression

After induction, the animals were extubated and awakened with the gradual reduction of anesthesia and mechanical ventilation. The spontaneously breathing septic and sham-operated animals were placed in a test cage and observed for visible signs of sepsis progression, and a blood sample was taken 6 h after sepsis induction. The animals in both groups received 15 mL kg^−1^h^−1^ crystalloid iv at 6 h and 12 h after sepsis induction to maintain fluid balance (Ringerfundin^®^, B. Braun, Melsungen, Germany), while analgesia was performed with nalbuphine iv (0.2 mg kg^−1^; Orpha-Devel Handels und Vertriebs GmbH, Austria) through the jugular vein.

#### Stage 4. Invasive Hemodynamic Monitoring, Sampling and Severity Scoring

The animals were re-anesthetized 15 h after sepsis induction with an iv mixture of ketamine (2.5 mg kg^−1^) and xylazine (0.5 mg kg^−1^), and anesthesia was maintained with a continuous infusion of propofol (6 mg kg^−1^h^−1^ iv; Fresenius Kabi, Bad Homburg, Germany), midazolam (1.2 mg kg^−1^h^−1^; Torrex Chiesi Pharma, Vienna, Austria) and fentanyl (0.02 mg kg^−1^h^−1^; Richter Gedeon, Budapest, Hungary). The adequacy of the depth of anesthesia was assessed by monitoring the jaw tone and the pain reaction of the hind leg regularly. After reintubation, mechanical ventilation was started, the settings (RR: 10–12 min^−1^; TV: 10 mL kg^−1^; PEEP: 4–5 cmH_2_O; FiO_2_: 21%) were checked and adjusted based on arterial and venous blood gas values (pH: 7.35–7.45; arterial partial carbon dioxide pressure (PaCO_2_): 35–45 mmHg (4.6–5.9 kPa), PaO_2_/FiO_2_ ratio > 400) during the monitoring period. After induction of anesthesia, the right femoral artery was dissected free using an aseptic technique, and a thermistor-tip transpulmonary thermodilution catheter (PiCCO, PULSION Medical Systems SE, Munich, Germany) was placed in the right femoral artery for invasive hemodynamic monitoring and core temperature measurement. A urinary catheter was placed surgically in the bladder via an inguinal incision to measure hour diuresis (mL kg^−1^ h^−1^).

The monitoring period for SOFA scoring started 16 h after induction on sepsis. Hemodynamic measurements, blood gas analysis and an assessment of organ failure were performed hourly between 16 and 24 h of the experiments. Cardiac output (CO) and cardiac index (CI) were measured by transpulmonary thermodilution analysis, HR and MAP were monitored with the pressure sensor of a transpulmonary thermodilution system. All hemodynamic parameters were indexed for body surface area or body weight. Central venous pressure (CVP) was measured via a central venous catheter at the same times as the other hemodynamic variables. Blood gases were analyzed with a cooximetry blood gas analyzer (Cobas b 123, Roche Ltd., Basel, Switzerland) simultaneously every hour.

Oxygen delivery (DO_2_) = CO × [(1.38 × Hb × SaO_2_) + (0.003 × PaO_2_)], oxygen consumption (VO_2_) = CO × [(1.38 × Hb × (SaO_2_- SvO_2_)) + (0.003 × PaO_2_)] and oxygen extraction (ExO_2_) = DO_2_ ${\text{VO}}_{2}^{-1}$ values were calculated. The degree of respiratory failure was determined using the PaO_2_/FiO_2_ ratio. The vascular resistance index (SVRI) and stroke volume index (SVI) were calculated according to standard formulas (SVRI = MAP-CVP CO^−1^; SVI = CI HR^−1^).

Fluid resuscitation was started during the monitoring period with combinations of balanced crystalloid solutions (Ringerfundin^®^, Ringer-lactate^®^, Sterofundin G^®^, B. Braun, Melsungen, Germany) of 15 mL kg^−1^. Goal-directed fluid resuscitation was performed to achieve physiological SVI values (SVI: 35–45 mL beat^−1^ m^−2^). The volume and type of crystalloid infusion were guided by continuously monitored CVP (5–8 mmHg) pulse pressure variance (8–12%), extravascular lung water index (ELWI: 6–8 ml kg^−1^) and venous glucose (4.1–5.6 mmol L^−1^) values (19).

### Detection of Organ Functions, and Metabolic and Inflammatory Markers

Blood samples were taken from the central venous line and placed in precooled, EDTA-containing tubes, centrifuged (1,200 g at 4°C for 10 min) and stored at −70°C until assay. Liver dysfunction was assessed by measuring plasma bilirubin, aspartate aminotransferase (AST) and alanine aminotransferase (ALT) levels, whereas the extent of kidney injury was estimated by measuring plasma creatinine and albumin levels using a Roche/Hitachi 917 analyzer (F. Hoffmann-La Roche AG, Switzerland). The lactate level as an indicator of metabolic imbalance was measured from venous blood samples (Accutrend Plus Kit, Roche Diagnostics Ltd., Rotkreuz, Switzerland). All analyses were performed on coded samples in a blinded fashion. Plasma levels of TNF-α and IL-10 as well as bET and HMGB1 were determined from these samples using commercial ELISA kits (Cusabio Biotechnology Ltd., Wuhan, China and Biomedica Ltd., Vienna, Austria, respectively) according to manufacturer's instructions.

### Blood Cell Counts

Samples for platelet, white blood cell and red blood cell counts were placed in EDTA-coated tubes and analyzed within 4 h with an automated cell counter (based on electrical impedance, Sysmex XE2100, Japan).

### Blood Culture Analysis

Five mL blood samples were obtained from the right jugular vein of 14 randomly selected animals in the septic group at 18 h of the experiments with the aseptic technique and then transferred to aerobic and anaerobic media bottles. BD BACTEC Plus/Anaerobic/F bottles (Becton Dickinson, Hungary) were used in a qualitative procedure to identify microbial occurrence in the blood samples as another part of the microbial profile. Then, the pairs of blood culture bottles were immediately transferred to the microbiology laboratory for analysis. The bottles were incubated in a BD BACTEC™ FX blood-culturing instrument (Becton Dickinson, UK) until positive signaling from the system occurred. Bottles containing blood without bacteria were used and incubated for 24 h as a control measurement. Microorganisms were identified according to manufacturer's instructions in the MALDI-TOF MS (Bruker Daltonics, Germany), as described before (18).

### Calculation of pSOFA Scores

Similarly to the human SOFA and quick SOFA scores, we established two scoring systems for the pigs consisting of three or five domains of organ/organ system dysfunction parameters. The 3-domain-pSOFA (3D-pSOFA) score involves respiration, MAP and hour diuresis, which can be determined based on readings of online measurements, while the 5-domain-pSOFA (5D-pSOFA) score also includes additional laboratory tests (i.e. bilirubin and platelet count) (Table 1).

**Table 1:** The pig-specific Sequential Organ Function Assessment (pSOFA) scoring system with 3 or 5 domains (3D-pSOFA and 5D-pSOFA scores) to assess sepsis-induced organ dysfunction.

**pSOFA scores**		**Organ dysfunction**	**Parameters**	**Score values**					**Need for laboratory analytics**	**Detection time**
				**0**	**1**	**2**	**3**	**4**		
**5D-pSOFA score**	**3D-pSOFA score**	Respiration	PaO_2_/FiO_2_ ratio	≥400	<400	<300	<200	<100	No	Online (continuous)
		Cardiovascular	MAP (mmHg)	≥75	<75	<65	N <0.1	N>0.1	No	Online (continuous)
				*(≥70)*	*(<70)*	*(D <5 or Db)*	*(D > 5.1 or E ≤ 0.1 or N ≤ 0.1)*	*(D > 15 or E > 0.1 or N > 0.1)*		
		Renal	Urine output (mL kg^−1^ h^−1^)	>0.5	<0.5	<0.25	F <10	F > 10	No	Online (continuous)
				*-*	*-*	*-*	*(< ~0.25)*	*(>~0.13)*		
	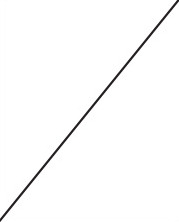	Liver	Bilirubin (μmol L^−1^)	<20	>20	>32	>101	>204	Yes	Hours
		Coagulation	Platelet Count (x10^9^ L^−1^)	≥200 *(≥150)*	<200 (<150)	<150 (<100)	<100 (<50)	<50 (<20)	Yes	Hours

*Arterial partial pressure of oxygen dissolved in the plasma (PaO_2_); fraction of inspired oxygen (FiO_2_); mean arterial pressure (MAP); N, norepinephrine; D, dopamine; Db, dobutamine; E, epinephrine; F, furosemide. Catecholamine doses are given as μg kg^– 1^ min^–1^ and furosemide doses as mg*.

*Human reference values (if different) are also shown (in italics). The 3D-pSOFA scoring system contains parameters which do not require laboratory diagnostics and can be evaluated continuously, thus allowing for closer monitoring. The 5D-pSOFA scoring system provides more information by assessing blood coagulation and liver function at the price of time-consuming laboratory tests*.

As a respiratory parameter, the PaO_2_/FiO_2_ ratio was used with threshold values similar to those in humans, but, in the case of MAP (used as a cardiovascular parameter), the blood pressure-lowering effect of continuous anesthesia and analgesia (10) was also taken into consideration. Therefore, MAP values above 75 mmHg were considered normal (SOFA = 0). Due to the relatively short observation period, the common first-line vasoactive drug norepinephrine was used for vasopressor therapy due to its efficacy, potency and safer dosing compared to dopamine (19). Norepinephrine was administered if MAP decreased to 55 mmHg and if it did not respond to at least 60 min of crystalloid resuscitation.

During 3D- or 5D-pSOFA scoring, urine output (and not serum creatinine level) was used as an indicator of renal dysfunction (Table 1). Furosemide (10 mg; iv) administration was initiated if urine output was low (<0.25 mL kg^−1^ h^−1^) despite the fluid administration or in the case of elevated ELWI (>10 mL kg^−1^). Repeated furosemide was administered if urine output and/or ELWI did not respond to the previous dose.

In addition to these parameters, the 5D-pSOFA score was also included (similarly to the human SOFA score) as well as platelet counts and plasma bilirubin values. Since pigs display a broader platelet count range than humans (10), values up to 200 10^9^ L^−1^ were considered normal (pSOFA scores *t* = 0 h; Table 1). We used the same threshold values for bilirubin as those used in human SOFA scoring. We also calculated the De Ritis ratio (AST/ALT) to detect liver damage and injury of non-liver cells (kidney, heart and muscle cells), but, ultimately, it was not used for scoring.

Before stage 4, we continuously evaluated the activity and alertness of the pigs to assess the well-being of the awake animals. We used a semi-quantitative 0–1 scoring system, but this neurological assessment was not incorporated into the pSOFA scoring system, as it was not considered to be equivalent to the human Glasgow Coma Score.

### Intravital Videomicroscopy

The Incident Dark Field (IDF) imaging technique (CytoCam Video Microscope System, Braedius Medical, Huizen, the Netherlands) was used for a non-invasive, contrast agent-free examination of the sublingual microcirculation. Cytocam-IDF imaging is optimized for visualization of hemoglobin-containing structures by illuminating the tissue surface with linearly polarized light and detecting the reflected light with a computer-controlled sensor (20, 21). Microcirculatory measurements were performed at *t* = 0, 16, 20 and 24 h, and the images were captured and recorded in six, 50-frame-long, high-quality video clips (spatial resolution 14 megapixels; temporal resolution 60 fps). Each video was recorded at separate locations of the sublingual area by the same investigator and saved as digital AVI-DV files to a hard drive. Every video clip was divided into four quarters and was determined by offline software-assisted analysis (AVA 3.0; Automated Vascular Analysis, Academic Medical Center, University of Amsterdam). The proportion of perfused vessels (PPV) was calculated as the ratio of the length of vessels with measurable flow to the length of all detected vessels (%) (22, 23).

### Statistical Analysis

Data analysis was performed with a statistical software package (SigmaStat for Windows, Jandel Scientific, Erkrath, Germany). Normality of data distribution was analyzed with the Shapiro–Wilk test. The Friedman analysis of variance on ranks was applied within groups. Time-dependent differences from the baseline for each group were assessed with Dunn's method. In this study, differences between groups were analyzed with the Kruskal–Wallis one-way analysis of variance on ranks, followed by Dunn's method. Median values and 75th and 25th percentiles are provided in the figures; *P* values <0.05 were considered significant. Correlations between two variables were examined using the Spearman Rank correlation coefficient (r); regression lines and 95% confidence intervals are given in the figures.

## Results

Despite the standardized sepsis induction protocol used in 27 pigs, eight experiments had to be excluded from the analysis for objective reasons. As noted before, five pigs were non-responders (non-septic) (Supplementary Table 1), showing higher values only in oxygen dynamic parameters and lower plasma albumin values compared to sham-operated animals between 18 and 24 h of the experiment. Another three pigs acted as over-responders with a fulminant septic reaction. These animals were humanely terminated (between 6 and 15 h of the study). The severity of their condition was characterized by signs of acute respiratory and/or circulatory failure, early (6 h) rise in lactate, bilirubin and hemoglobin levels, and steep decreases in venous oxygen saturation and albumin levels (Supplementary Table 2). Data from the non-septic and terminated animals are not included in further results.

### Changes in pSOFA Scores

**Figure 2:**
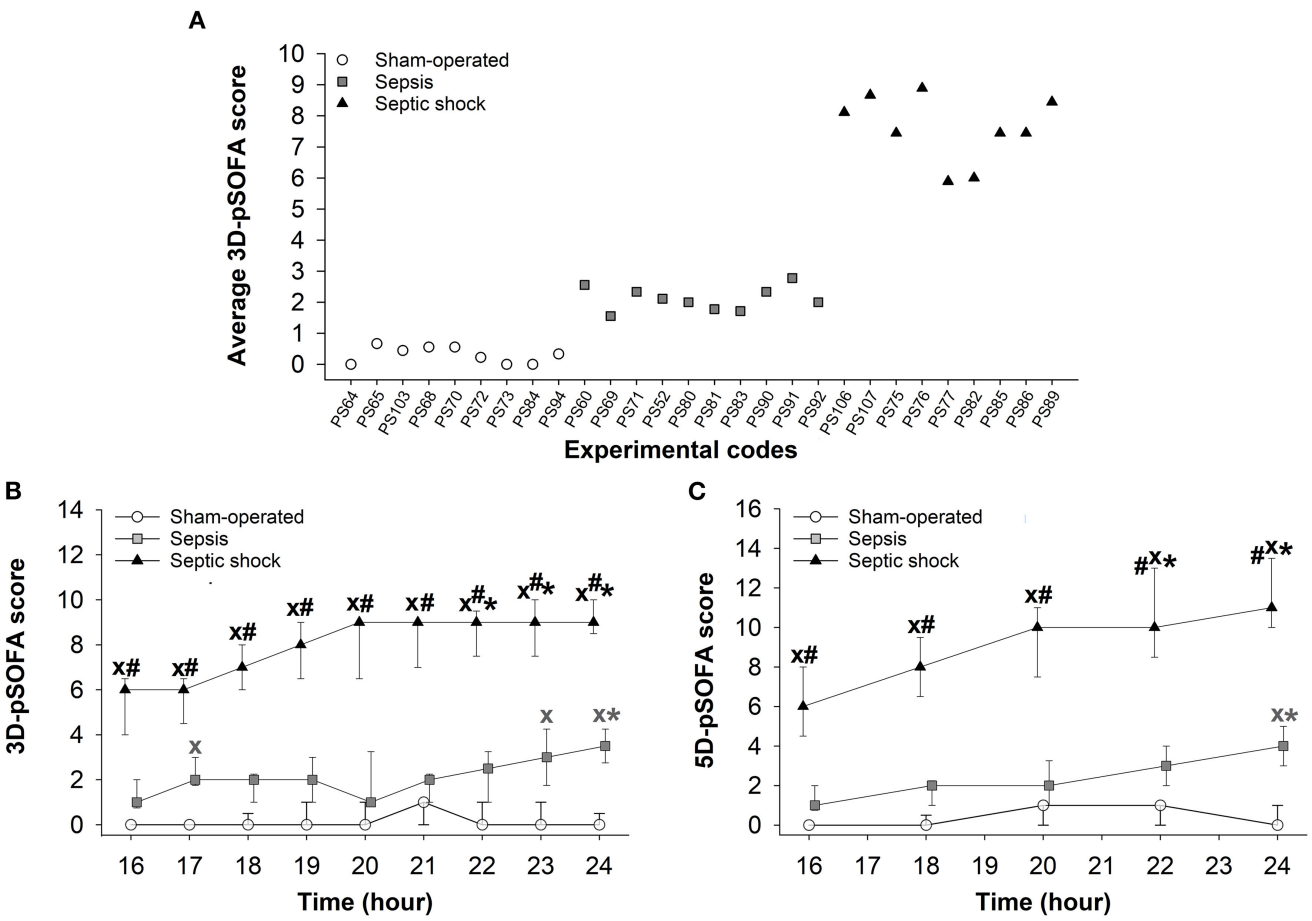
Average values for basic pSOFA scores in individual animals during the 8-h invasive monitoring period (during *t* = 16–24 h) **(A)** in the sham-operated group (open circles) and the sepsis (gray square) and septic shock subgroups (black triangle). Changes in 3D-pSOFA **(B)** and 5D-pSOFA **(C)** values as a function of time in the sham-operated group and the sepsis and septic shock subgroups. The plots demonstrate the median values and the 25th (lower whisker) and 75th (upper whisker) percentiles. ^X^*P* < 0.05 vs. sham-operated group; ^#^*P* < 0.05 vs. sepsis group; *P < 0.05 vs. *t* = 16 h.

In the septic animals, the average 3D-pSOFA scores ranged from 1 to 3, while the score reached values between 5 and 9 in the case of septic shock (Figure 2A). In the septic shock subgroup, significantly higher 3D-pSOFA and 5D-pSOFA scores were detected than those in the sham-operated group and sepsis subgroup throughout the 8-h observation period (Figures 2B,C). A significant difference was observed between the sham-operated and septic groups at 24 h after the sepsis induction for both scores. As compared to *t* = 16 h, a temporal deterioration in the condition of the animals with septic shock was evident in the last 2–3 h of the experiments with both scores.

### Changes in Different Components of pSOFA Scores and in Biomarkers of Organ Dysfunction

**Figure 3:**
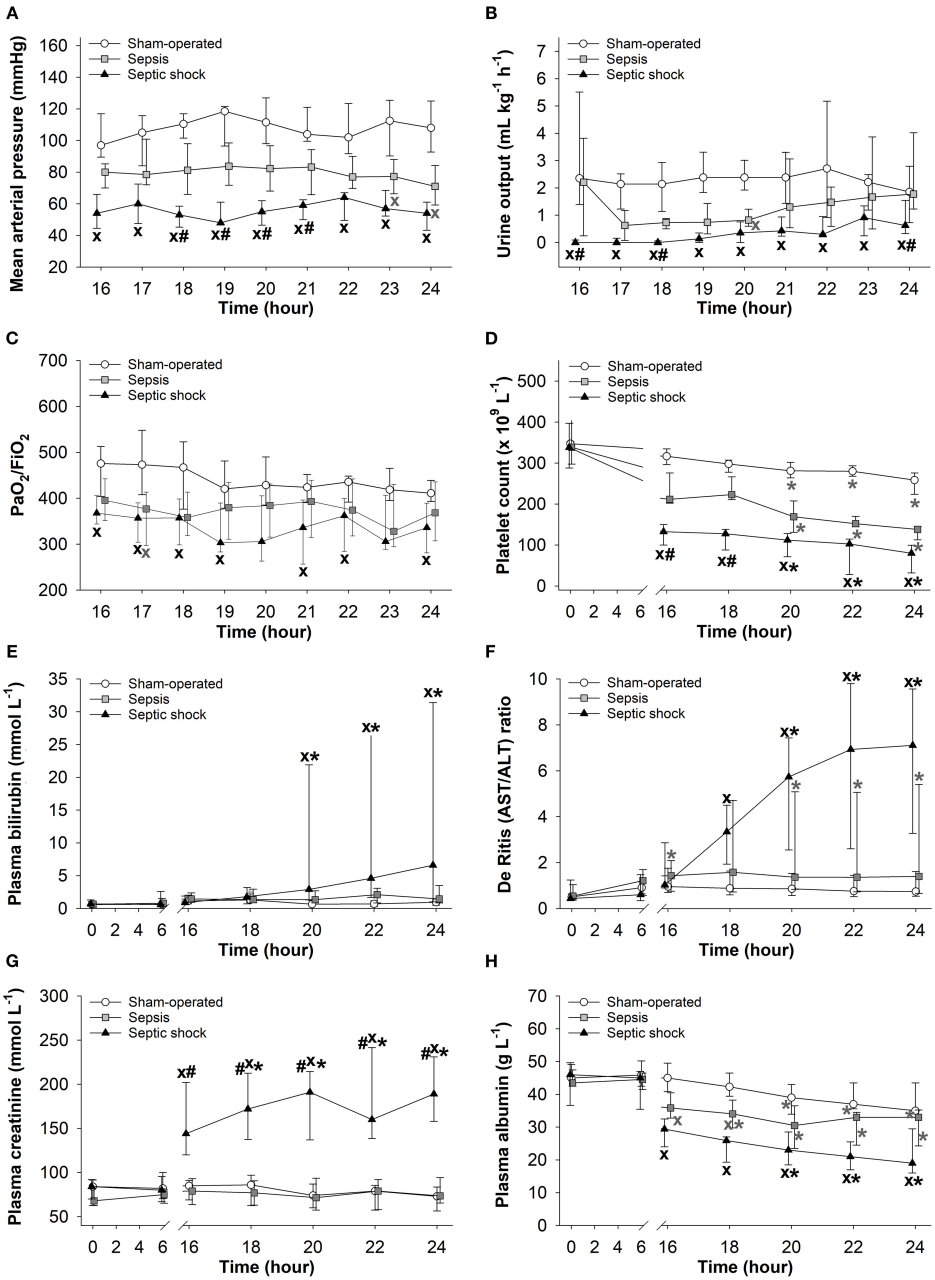
Changes in mean arterial pressure **(A)**, urine output **(B)**, PaO_2_/FiO_2_ ratio **(C)**, platelet count **(D)**, plasma bilirubin **(E)**, De Ritis (AST/ALT) ratio **(F)**, plasma creatinine **(G)**, and plasma albumin levels **(H)** in the sham-operated group (open circles) and the sepsis (gray square) and septic shock subgroups (black triangle). The plots demonstrate the median values and the 25th (lower whisker) and 75th (upper whisker) percentiles. ^X^*P* < 0.05 vs. sham-operated group; ^#^*P* < 0.05 vs. sepsis group; **(A–D)** *P < 0.05 vs. *t* = 16 h; **(E–H)** *P < 0.05 vs. *t* = 0 h.

Significantly lower MAP, urine output and platelet count values were evidenced in animals with septic shock than those seen in sham-operated animals during the entire 8-h observation period (Figures 3A,B,D). A temporary hypotension also developed in the sepsis subgroup in the last two hours (Figure 3A). Deteriorations in urine output and in PaO_2_/FiO_2_ ratio reached a similar extent in the sepsis and septic shock subgroups at most examined time points (Figures 3B,C). A progressive decrease in platelet count was observed in all groups during the last 3 h of the study period, with the lowest values in the septic shock subgroup (Figure 3D). As compared to the sham-operated animals, only the septic shock subgroup showed significant elevations in plasma bilirubin concentrations and in the (hepatic and non-hepatic cell degradation marker) AST/ALT ratio (De Ritis ratio), which occurred during the last 3–4 hours of the study (Figures 3E,F). Plasma creatinine levels were also higher in the septic shock subgroup than in the other groups (Figure 3G), and we only found a negative correlation between plasma creatinine levels and the decreased urine output in the septic shock group (*r* = −0.352) (Supplementary Figure 1). A progressive decrease in plasma albumin levels was observed in all the groups. As compared to sham-operated animals, septic shock was always associated with significantly lower albumin values, and the animals in the sepsis subgroup also showed temporarily lower albumin values during *t* = 16–24 h of the study (Figure 3H).

### Blood Cell Counts

**Figure 4:**
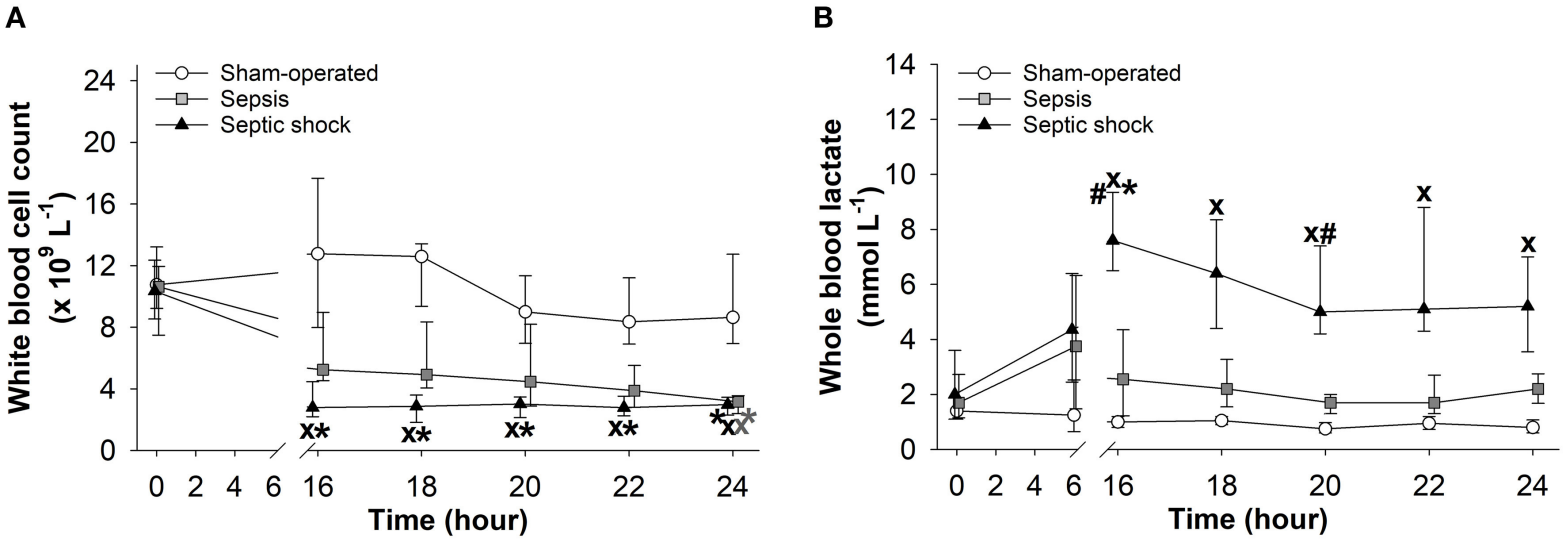
Changes in white blood cell count **(A)** and whole blood lactate levels **(B)** in the sham-operated group (open circles) and the sepsis (gray square) and septic shock subgroups (black triangle). The plots demonstrate the median values and the 25th (lower whisker) and 75th (upper whisker) percentiles. ^X^*P* < 0.05 vs. sham-operated group; ^#^*P* < 0.05 vs. sepsis group; *P < 0.05 vs. *t* = 0 h.

White blood cell counts did not change in the sham-operated groups, while significant leucopenia developed after 16 h of sepsis induction in both sepsis-inoculated groups (Figure 4A). The red blood cell counts did not change in any of the groups studied (data not shown). Whole blood lactate showed some degree of elevation at 6 h after induction in both septic groups, but it only reached significantly higher values in the septic shock group, which persisted during the entire period *t* = 16–24 h (Figure 4B).

### Hemodynamic Changes

**Figure 5:**
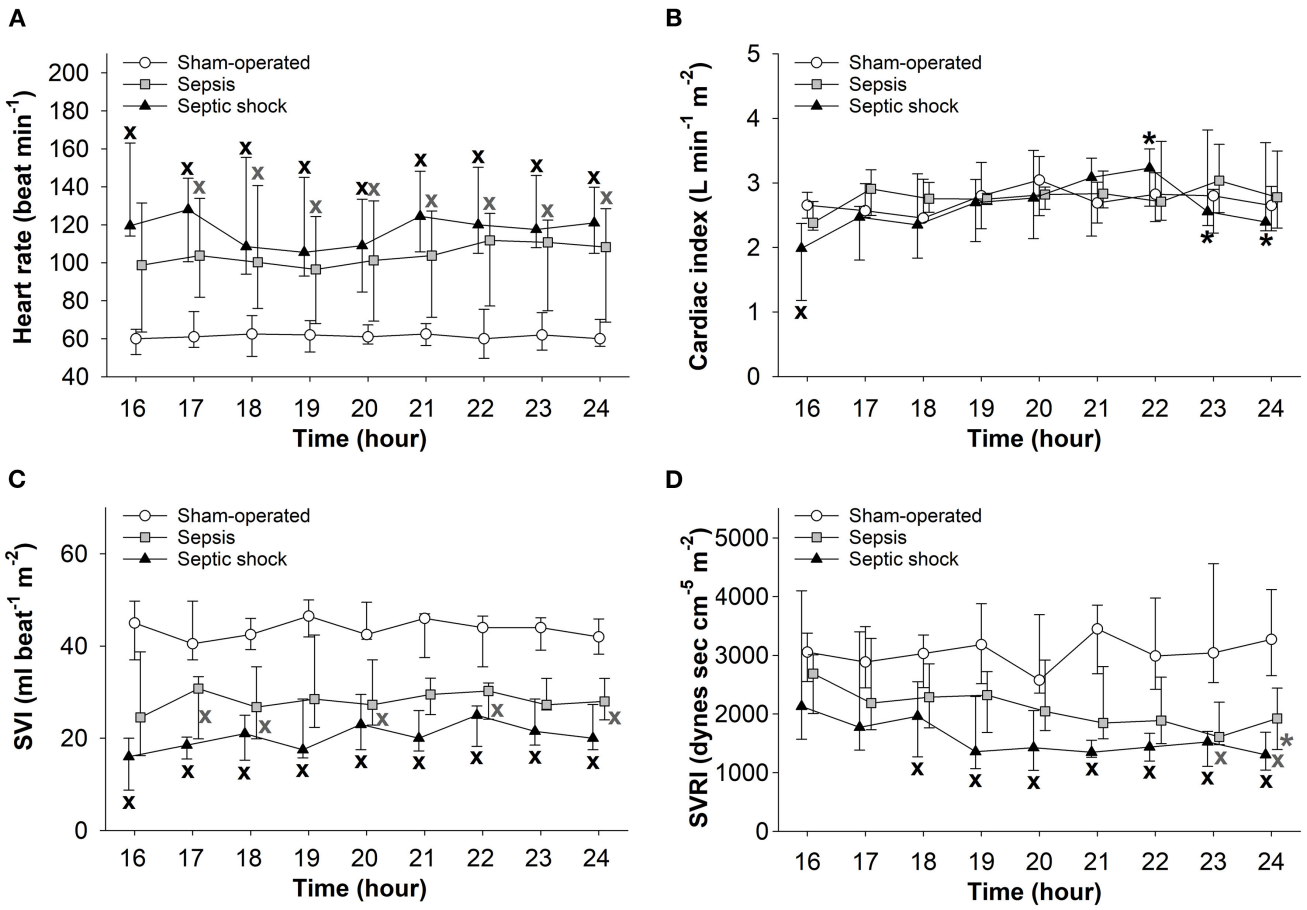
Changes in heart rate **(A)**, cardiac index **(B)**, stroke volume index (SVI) **(C)**, and systemic vascular resistance index (SVRI) **(D)** in the sham-operated group (open circles) and the sepsis (gray square) and septic shock subgroups (black triangle). The plots demonstrate the median values and the 25th (lower whisker) and 75th (upper whisker) percentiles. ^X^*P* < 0.05 vs. sham-operated group; *P < 0.05 vs. *t* = 16 h.

In both sepsis-inoculated groups, a significant increase in HR and a significant decrease in stroke volume were observed during the entire examination period and temporary decreases also occurred in systemic vascular resistance (Figures 5A,C,D). In the septic shock group, cardiac index also deteriorated during the last two hours of the experiments (Figure 5B).

### Changes in Oxygen Dynamics and Microcirculation

**Figure 6:**
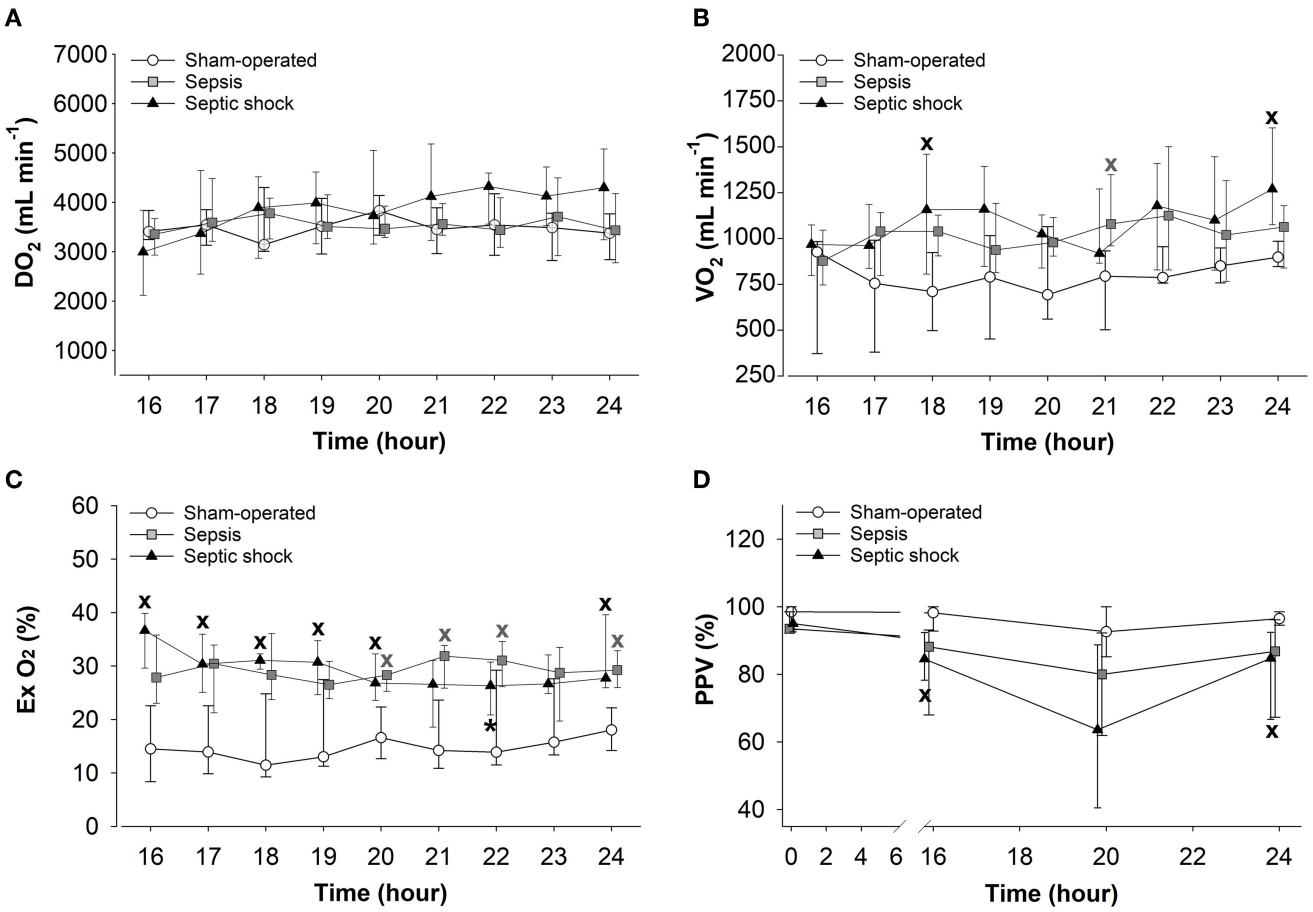
Changes in oxygen delivery (DO_2_) **(A)**, oxygen consumption (VO_2_) **(B)**, oxygen extraction (Ex O_2_) **(C)**, and the proportion of perfused vessels (PPV) **(D)** in the sham-operated group (open circles) and the sepsis (gray square) and septic shock subgroups (black triangle). The plots demonstrate the median values and the 25th (lower whisker) and 75th (upper whisker) percentiles. ^X^*P* < 0.05 vs. sham-operated group; *P < 0.05 vs. *t* = 16 h.

The 24-h sepsis progression markedly affected the oxygen dynamic parameters. Although no differences were observed in DO_2_ (Figure 6A), significantly increased VO_2_ values were measured in septic shock subgroup at 18 and 24 h compared to the sham-operated animals (Figure 6B). Both sepsis-challenged groups showed a significantly elevated degree of oxygen extraction (Figure 6C) and septic shock was associated with significantly deteriorated microvascular perfusion (reduced PPV values) in the sublingual mucosa (Figure 6D).

### Microbial Features of the Inducer Inoculum

Different CFU ranges in the sepsis-inducer inoculum caused a different degree of sepsis severity. The retrospective microbiological analysis demonstrated that the microbial concentration of the inoculum ranged between 6.2 × 10^5^ and 1.6 × 10^10^ CFU and that concentrations under 1.34 × 10^7^ CFU did not result in a septic reaction in the 24 h period of the experiment (*n* = 5, non-responder subgroup). Concentrations above 4 × 10^8^ CFU increased the likelihood of sepsis and septic shock, while CFU values above 8 × 10^9^ resulted in a devastating condition with quick deterioration in animal well-being (*n* = 3, 8 × 10^9^-1.6 × 10^10^ CFU). The most common microorganisms found in the feces suspension were *Escherichia coli* and *Klebsiella pneumoniae*, which generally cause Gram-negative sepsis in humans. The occurrence of *Escherichia coli* was 100% in the fecal samples. We also found several *Lactobacilli* species, which are indicators of normal microbiota but can also cause sepsis under dysbiotic conditions or when the intestinal epithelial layer is injured (Supplementary Table 3).

### Relationship Between Microbiological Concentrations and pSOFA Scores

**Figure 7:**
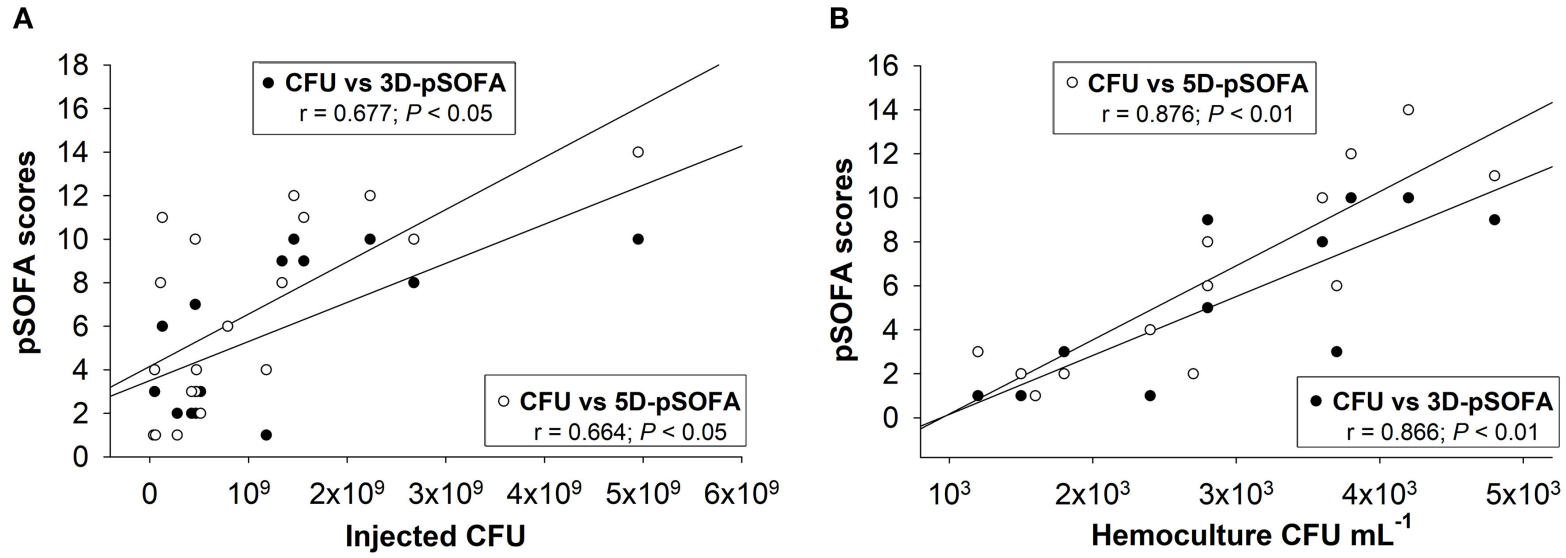
Correlation between the concentration of injected microorganism in feces and the pSOFA score values **(A)**. Correlation between the microbial concentration in the hemoculture and the pSOFA score values **(B)**.

The correlation between the severity of organ dysfunction and the concentration of injected microorganisms or the presence of microbes in the blood samples were analyzed further in septic animals. There was a moderate, significant positive correlation between the inoculum CFUs and the 3D-pSOFA (*r* = 0.677) and the 5D-pSOFA score values (*r* = 0.664; *n* = 19) (Figure 7A). The concentration of microbes in the blood culture showed a strong, significant correlation (*r* = 0.866, *P* < 0.001) with both the 3D-pSOFA and the 5D-pSOFA score values (*n* = 14) (Figure 7B).

### Changes in Plasma Levels of Inflammatory Biomarkers

**Figure 8:**
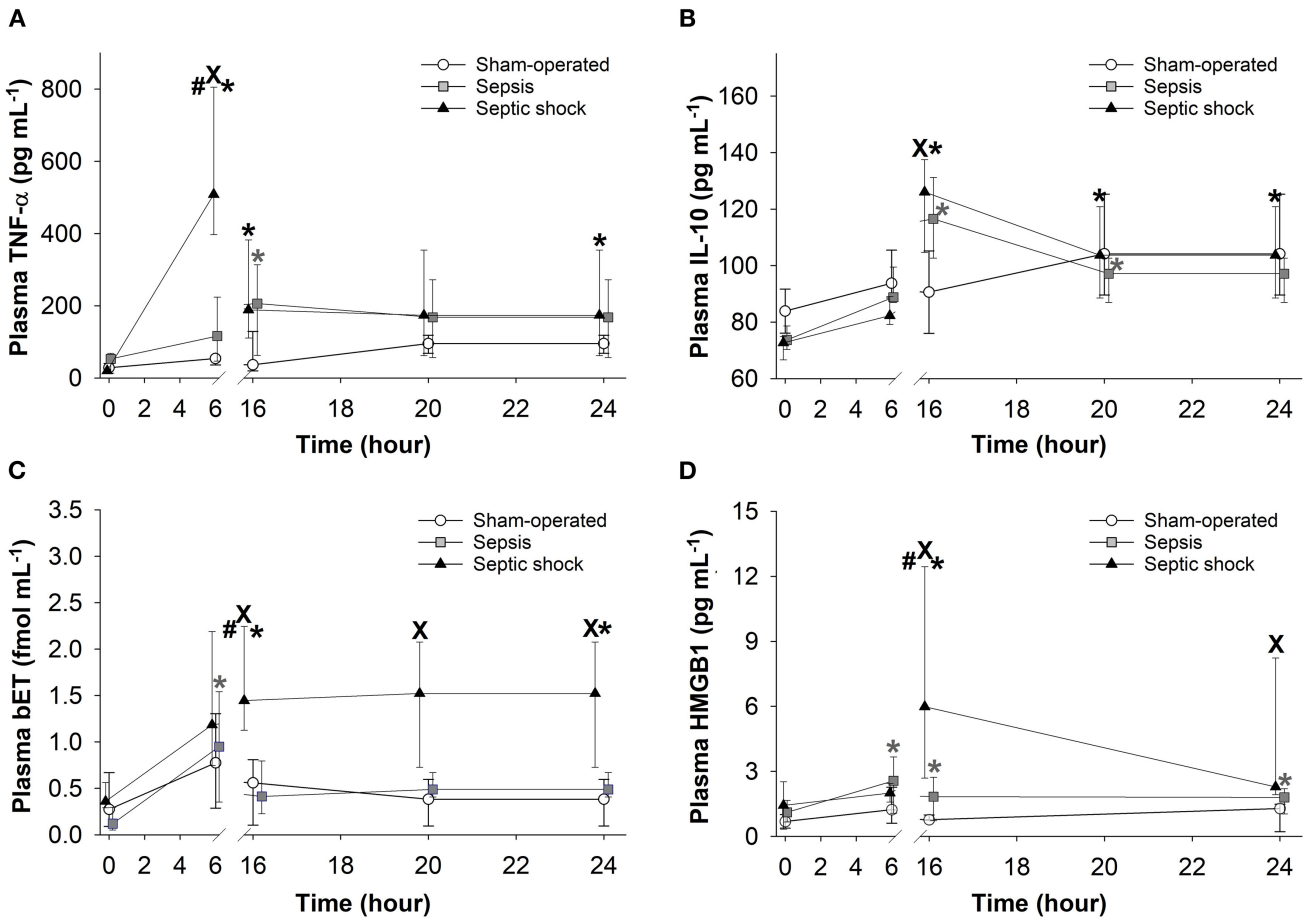
Changes in plasma tumor necrosis factor alpha (TNF-α) **(A)**, interleukin-10 (IL-10) **(B)**, big endothelin (bET) **(C)**, and high-mobility group box 1 protein (HMGB1) **(D)** levels in the sham-operated group (open circles) and in the sepsis (gray square) and septic shock subgroups (black triangle). The plots demonstrate the median values and the 25th (lower whisker) and 75th (upper whisker) percentiles. ^X^*P* < 0.05 vs. sham-operated group; ^#^*P* < 0.05 vs. sepsis group; *P < 0.05 *t* = 0 h.

As compared to the baseline, plasma TNF-α concentration peaked at 6 h after sepsis induction, and this significant elevation persisted during the entire period of invasive hemodynamic monitoring in the septic shock subgroup (Figure 8A). In this phase, similar TNF-α levels were detected in both septic groups. Plasma levels of IL-10 also showed a peak at 16 h in both septic groups (Figure 8B). Plasma levels of bET and HMGB1 only increased in the septic shock subgroup at 16–24 h post-inoculation (Figures 8C,D).

### Correlation Between Plasma Biomarkers and pSOFA Scores

**Figure 9:**
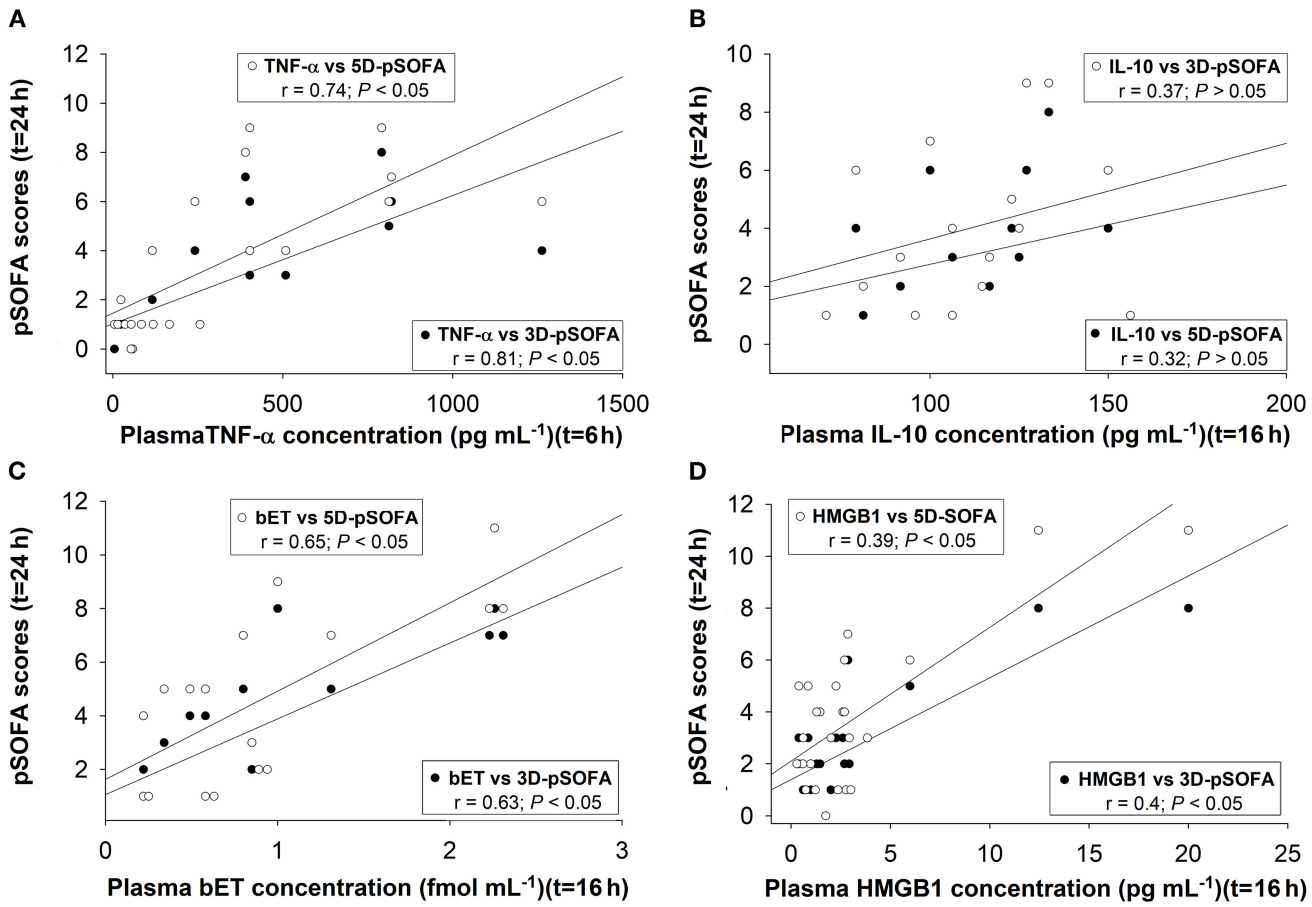
Correlations between plasma concentrations of TNF-α at 6t = h **(A)**, interleukin-10 (IL-10) **(B)**, big endothelin (bET) **(C)**, high-mobility group box 1 protein (HMGB1) at *t* = 16 h **(D)**, and 3D-pSOFA and 5D-pSOFA scores at *t* = 24 h.

We found significant correlations between the 6-h TNF-α (Figure 9A), the 16-h bET (Figure 9C) and the 16-h HMGB1 levels (Figure 9D) as well as the 24-h 3D- and 5D-pSOFA scores. However, we did not find any correlation between the 16-h IL-10 values and the 24-h 3D- or 5D-pSOFA scores (Figure 9B).

## Discussion

### Overview of the Experimental Model

Here we present a porcine model of polymicrobial, intraabdominal sepsis with clinically relevant hemodynamic responses, a laboratory profile, inflammatory biomarkers and bacteremia. We also introduce a simple and a more complex pSOFA scoring system to assess organ dysfunction according to the Sepsis-3 criteria. Further, we have established the predictive significance of plasma biomarkers indicative of inflammation, tissue hypoxia or necrosis to diagnose late-onset organ damage. It should be added that the protocol was designed according to the recommendations of the MQTiPSS guidelines with respect to infection types, study design, assessment of organ failure, proper analgesia, fluid resuscitation and humane endpoints (3).

This setup combines the advantages of conscious, anesthetized *in vivo* models, while adhering to ethical standards on the use of animals for scientific purposes. After a relatively short (~45 min) general anesthesia with limited instrumentation, sepsis develops in a conscious state. Non-invasive monitoring with adequate postoperative analgesia and restrictive fluid therapy is then maintained in the first 15 h of progression. After re-anesthesia, animals can safely be subjected to extensive invasive instrumentation, with complex monitoring and resuscitation for at least 8 h. A minor limitation is that a design with an observation time of this length or longer requires age- and time-matched sham-operated controls to avoid the distorting effect of self-control comparisons.

The majority of large animal models of bacterial sepsis involve administration of living bacterial monocultures (such as *E coli* and *S aureus*) or polymicrobial fecal inoculum (8–10, 24, 25). Nevertheless, in terms of clinical relevance, polymicrobial autofeces induction is probably more reliable then bacterial monocultures. Although the predominant microorganisms in feces are variable, the inflammatory host response caused by the invading pathogens might be identical to the clinical situation (9). Here we also confirmed that the standardized, autologous inoculum resulted in a predominantly *E coli*-characterized bacteremia, similarly to many clinical and preclinical observations (9, 26).

Organ supportive therapies (respiratory, fluid, diuretic and vasopressor or inotropic) are used as in the ICUs. These individualized treatments began 16 h after the insult in a goal-directed manner according to the current condition of the animals and using recommendations similar to those for humans (27, 28). Fluid resuscitation was performed with balanced crystalloid solutions based on the current state of the animals to avoid metabolic (hypo- and hyperglycemia) or ion imbalance (29).

The hemodynamic and biochemical changes were severity-dependent, but the oxygen dynamic parameters differed less between the animals in the sepsis and septic shock groups within the observation period. The decreased SVRI, increased DO_2_ and VO_2_, and markedly increased ExO_2_ demonstrated a compensated hyperdynamic state in this model of progressive sepsis. In this line, sublingual microcirculation was reduced significantly in both septic groups, but an improving trend was observed in capillary perfusion rate, probably in association with the resuscitation therapies applied.

### Assessing Sepsis Severity Using 3-Domain and 5-Domain-pSOFA Scoring Systems

In this model, the animals presented an almost human-like septic reaction, including elevated lactate level, hypotension, hypovolemia and multi-organ dysfunction caused by a polymicrobial infection. Furthermore, despite the highly standardized inducer conditions, each of the inoculated animals presented with various, individual reactions. Therefore, we divided the treated animals into non-septic, septic, septic shock and fulminant sepsis subgroups, but only the data from the septic and septic shock subgroups were examined in detail. The severity of organ damage ranged from less severe to septic shock, and a numerical characterization of these individual reactions was achieved using a pig-specific scoring system. It should be added that the human SOFA score has already been applied in unchanged form (8) or in a modified version (10) in preclinical porcine sepsis studies. Here we propose the use of 3- and 5-domain froms of pSOFA to quantify organ damage comprehensively. Like human quick scoring, the simplified pSOFA score is suitable for “bedside” evaluation of the severity of sepsis with basic parameters (MAP, PaO_2_/FiO_2_ ratio and urine output), which does not require time-consuming laboratory testing, thus allowing for a quick, online evaluation of the status of organ function. The extended scoring and measurement of the components of the 5D-pSOFA score (platelet counts and bilirubin levels) require laboratory testing and thus may not capture the dysfunction of individual organ systems promptly, but, with higher offline resolution, it provides more information on sepsis progression. This possibility is supported by the fact that it was only the 5D-pSOFA score that indicated sepsis progression in the “non-septic” group as compared to the sham-operated group (Supplementary Table 1).

In our protocol, the scoring systems were applied 16 h after induction. However, it has already been shown that human-like SOFA scores can be used at earlier time points in pigs (10). Park et al. reported on changes in relevant parameters of sepsis progression (including some of the SOFA parameters) in anesthetized pigs approx. after 6–12 h after induction (9). Similar changes were observed in conscious pigs with a longer follow-up (8, 10). Based on these references, we hypothesized that the earliest signs of multi-organ failure could be detected by our pSOFA scoring system between 6 and 12 h.

### Calculating the Components of pSOFA Scoring System

Among the elements of the human SOFA score, the PaO_2_/FiO_2_ ratio was used in its original form (30). Other elements, such as the cardiovascular score, can be affected by iatrogenic interventions or anesthesia. Since general anesthetics can be cardiodepressive and decrease systemic blood pressure due to reduced sensitivity of the baroreflex (12, 31, 32), a higher threshold value for the MAP (75 mmHg) was defined to indicate the beginning of cardiovascular dysfunction.

In the case of the renal system, plasma creatinine levels increased significantly and only showed a correlation with urine output in the septic shock group. The increase of the creatinine plasma level was insufficient to demonstrate renal dysfunction in the sepsis group, while a decreased tendency in urine output was evident here. This suggests that urine output is a more sensitive indicator of early renal injury after the septic insult in this experimental setup. Since creatinine is a measure of renal perfusion/filtration, a large percentage of the kidney mass has to be dysfunctional before a rise in creatinine concentration is seen (8). Therefore, we recommend monitoring urine output to assess renal damage in relevant preclinical studies. The additional decrease in albumin level can also provide useful information on renal and liver function (33).

In addition, according to the latest literature data, novel cell cycle arrest biomarkers and neutrophil gelatinase-associated lipocalin can indicate kidney dysfunction even earlier than urine output and creatinine (34).

Plasma bilirubin is an accepted biomarker for assessment of liver dysfunction in large animal studies. Nevertheless, significant changes occurred in later phases, between 20 and 24 h, and only in a few animals with septic shock. This suggests that liver damage develops relatively late and depends on the severity of insult. In septic human patients, significantly increased bilirubin levels within 72 h indicate severe hepatic insufficiency and a high risk of mortality (35). In previous reports, the human range of plasma bilirubin was used in pigs with minor modifications (10). In addition, AST, ALT and creatine kinase data are often used to diagnose hepatic dysfunction in preclinical sepsis models. The De Ritis ratio (as AST/ALT ratio) is another commonly used marker of hepatic cell degradation. Increasing ALT level is a relatively specific sign of hepatocellular damage, while separated elevation of AST is usually due to injury of liver and non-liver cells (kidney, heart and muscle cells) as well (36). Therefore, we propose the use of the De Ritis ratio for the assessment of sepsis-related liver damage in short-term experimental designs.

A decreased platelet count is a measure of the activation of the coagulation system in human SOFA scoring; it has previously been used in other porcine sepsis studies (8–10, 37). However, pigs are normally hypercoagulable as compared to other species, including humans, and the normal platelet counts in pigs fall on a much broader range as compared to humans (38). Therefore, we defined higher platelet count categories for the 5D-pSOFA scoring to assess the presence of coagulation dysfunction (see Table 1). In our model, platelet number change seems to be a sensitive and early marker of severity of the septic process. It should be noted, however, that platelet activation easily occurs in pigs, especially when blood samples are collected through catheters, and the mechanical contact increases the chances of aggregation (38).

### Examining the Relationship Between Microbiology and Sepsis Severity

Quantitative microbiological analysis of the fecal inoculum showed that the onset and progression of sepsis–septic shock requires a critical number of germs in the induction suspension. We also examined the relationship between the degree of microbiological invasion and the host response leading to organ dysfunctions characterized by 3D- or 5D-pSOFA scoring. The initial microbial concentration of the inducer inoculum was moderately associated with the severity of organ dysfunction, while the concentration of microbes in the blood showed a much stronger correlation. Although the predominant microorganism in a fecal sample is variable, the inflammatory response of the host to the invading pathogens might be consistent, which is identical to the clinical situation (9). Nevertheless, it is important to note that individual microbiological diversity can contribute to the severity statuses observed in our experimental animals, which can complicate standardization. Therefore, frequent preliminary microflora testing may also facilitate the standardization of an autologous fecal inoculum-induced sepsis model.

### Assessing the Prognostic Value of Plasma Biomarkers in Sepsis

The pathogenesis of sepsis involves the release and activation of hundreds of mediator molecules, cytokines, acute phase proteins and stress hormones, which can be considered prognostic biomarkers (39) in animal models and human clinical studies as well (14). In our study, an early release of TNF-α at 6 h was noted, while the time-dependent kinetics of other detected biomarkers (IL-10, bET and HMGB1) were consistent with sepsis progression. Moreover, the dynamics of all the inflammatory mediators under examination showed significant differences between the sepsis and septic shock groups. Therefore, early (6 h) detection of plasma TNF-α and a somewhat late elevation of bET and HMGB1 levels may indicate the probability of septic shock-linked organ dysfunction quantified by the 3D- and 5D-pSOFA scores as well. Hence, we suggest that these biomarkers may have a potentially predictive importance in experimental sepsis.

### Study Limitations

This study has certain limitations. First, we used healthy young adult pigs which lacked comorbidities commonly associated with human sepsis. Second, the Glasgow Coma Scale used to quantify the damage to the central nervous system cannot be employed. Third, antibiotic treatment was not applied, since there was not enough monitoring time to evaluate the effect of the antibiotics on the blood culture results (which are available after 24–48 h, while a non-specific antibiotic treatment can be ineffective in 30% of cases), and knowledge of the untreated microbiological profile was an important point to consider in these experiments (40). Controlling the efficacy of antibiotic therapy by detecting the plasma procalcitonin level would be an alternative option, but this approach also requires a longer experimental protocol (41).

## Conclusion

We have described a porcine model of polymicrobial, intraabdominal sepsis in detail. The methodology combines the advantages of conscious and anesthetized studies, and mimics human sepsis and multi-organ failure very closely. The host responses were quantified with modified human SOFA-like 3D- and 5D-pSOFA scoring systems. 3D-pSOFA scoring is suitable for evaluation of the cardiovascular, pulmonary and renal dysfunctions online without laboratory biochemical testing, while the use of the 5D-pSOFA score improves reproducibility (with the extended assessment of coagulation and hepatic dysfunction parameters) and provides an alternative endpoint instead of mortality. In this context, sepsis and septic shock can also be well-distinguished. We thus propose that the standardization of large animal studies with the pSOFA scoring systems will make different models comparable, thus reducing the gap between preclinical and clinical outcomes.

## Data Availability Statement

The raw data supporting the conclusions of this article will be made available by the authors, without undue reservation.

## Ethics Statement

The animal study was reviewed and approved by National Scientific Ethical Committee on Animal Experimentation (National Competent Authority of Hungary; approval number: V/175/2018).

## Author Contributions

AR, BZ, ST, DÉ, LB, LJ, RF, GV, LB, and JK performed experiments and wrote the manuscript. ST, ZB, and JS performed microbiological examinations. MP and AS prepared figures. KB, AS, MB, and JK supervised and edited the manuscript. All authors read and approved the manuscript.

## Funding

Research grant from the National Research Development and Innovation Office, NKFI K120232.

## Conflict of Interest

The authors declare that the research was conducted in the absence of any commercial or financial relationships that could be construed as a potential conflict of interest.

## Publisher's Note

All claims expressed in this article are solely those of the authors and do not necessarily represent those of their affiliated organizations, or those of the publisher, the editors and the reviewers. Any product that may be evaluated in this article, or claim that may be made by its manufacturer, is not guaranteed or endorsed by the publisher.

## Supplementary Material

Supplementary Figure 1 Correlation between plasma creatinine concentration and urine output in the sepsis **(A)** and septic shock **(B)** subgroups.

Supplementary Table 1A Changes in the components and score values of the 3D-pSOFA and 5D-pSOFA scoring systems in the sham-operated (*n* = 9) and non-septic animals (*n* = 5) during 16 and 24 h of the monitoring period.

Supplementary Table 1B Changes in the hemodynamics in the sham-operated (*n* = 9) and non-septic animals (*n* = 5) during 16 and 24 h of the monitoring period.

Supplementary Table 1C Changes in the oxygen dynamics, microcirculation and white blood cell count in the sham-operated (*n* = 9) and non-septic animals (*n* = 5) during the monitoring period.

Supplementary Table 1D Changes in markers of organ dysfunction in the sham-operated (*n* = 9) and non-septic animals (*n* = 5) during the monitoring period.

Supplementary Table 2 Changes in the parameters of terminated animals (with fulminant septic reaction) at *t* = 0 and 6 h.

Supplementary Table 3 Microbial composition of the sepsis-inducing inoculum expressed as the incidence of the current strain per total number of samples (%).
