# Supplementary figures and images for: A Porcine Sepsis Model With Numerical Scoring for Early Prediction of Severity
Source: Front Med (Lausanne). 2022 May 9;9:867796. doi: 10.3389/fmed.2022.867796 (PMC9125192; doi:10.3389/fmed.2022.867796)

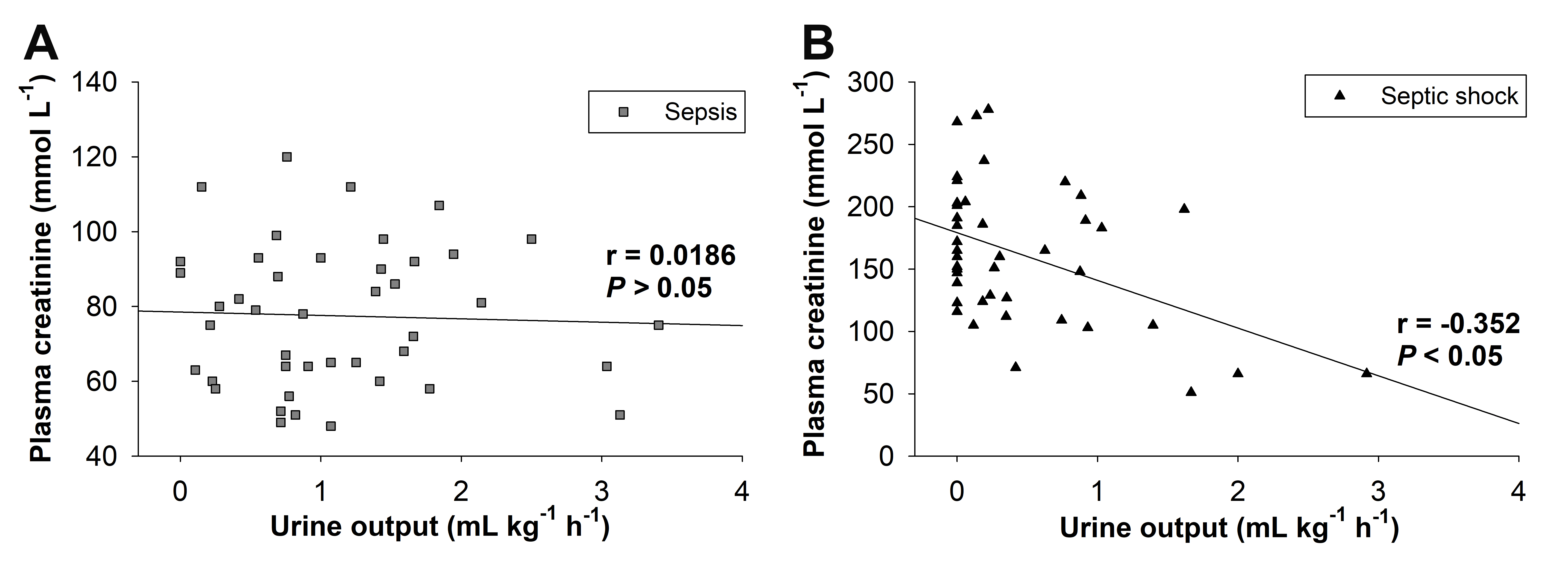

Supplement: Supplementary Figure 1 — Correlation between plasma creatinine concentration and urine output in the sepsis (A) and septic shock (B) subgroups. [file Image_1.TIF]
