# Supplementary material for: A Porcine Sepsis Model With Numerical Scoring for Early Prediction of Severity
Source: Front Med (Lausanne). 2022 May 9;9:867796. doi: 10.3389/fmed.2022.867796 (PMC9125192; doi:10.3389/fmed.2022.867796)
Supplement: Supplementary Table 1A — Changes in the components and score values of the 3D-pSOFA and 5D-pSOFA scoring systems in the sham-operated (n = 9) and non-septic animals (n = 5) during 16 and 24 h of the monitoring period. [file Table_1.docx]

Supplemental digital content – Table 1A Changes in the components and score values of the 3D-pSOFA and 5D-pSOFA scoring systems in the sham-operated (n=9) and non-septic animals (n=5) during 16 h and 24 h of the monitoring period.

|  |  | Median *(25-75% percentiles)* | | | | |
| --- | --- | --- | --- | --- | --- | --- |
| **Parameters** | **Group** | **16 h** | **18 h** | **20 h** | **22 h** | **24 h** |
| **Mean arterial pressure** | Sham-operated | 97 *(89-117)* | 110 *(101-117)* | 111 *(98-127)* | 102 *(91-123)* | 108 *(92-125)* |
| (mmHg) | **Non-septic** | 91 *(80-106)* | 94 *(89-104)* | 94 *(86-105)* | 94 *(89-101)* | 89 *(84-100)* |
| **PaO_2_/FiO_2_** | Sham-operated | 475 *(404-513)* | 467 *(376-523*) | 428 *(385-490)* | 435 *(391-448)* | 410 *(392-438)* |
|  | **Non-septic** | 391 *(337-537)* | 421 *(334-463)* | 406 *(353-452)* | 374 *(348-423)* | 366 *(341-423)* |
| **Urine output** | Sham-operated | 2.35 *(1.39-5.5)* | 2.14 *(1.14-2.92)* | 2.38 *(1.98-3.01)* | 2.7 *(0.91-5.17)* | 1.85 *(0.72-2.79)* |
| (mL kg^-1^ hr^-1^) | **Non-septic** | **0.85 *(0.32-1.78)*^x^** | **0.61 *(0.52-0.94)*^x^** | **0.68 *(0.48-1.68)*^x^** | **0.55 *(0.41-0.73)*^x^** | 0.73 *(0.6-1.29)* |
| **Bilirubin** | Sham-operated | 1.1 (0.7-2) | 1.3 *(1-2.2)* | 0.6 *(0.5-1.2)* | 0.7 *(0.3-1.7)* | 0.9 *(0.5-1.3)* |
| (mmol L^-1^) | **Non-septic** | 1.4 *(0.9-3.6)* | 1.2 *(0.9-1.4)* | 0.8 *(0.5-1.5)* | 0.9 (*0.65-1)* | 0.6 *(0.5-1.1)* |
| **Platelet count** | Sham-operated | 316 *(296-334)* | 297 *(277-307)* | 281 *(264-301)* | 280 *(267-293)* | **258 *(223-275)**** |
| (x10^9^ L^-1^) | **Non-septic** | 306 *(237-397)* | 298 *(203-376)* | 333 *(192-359)* | 281 *(175-299)* | **245 *(130-305)**** |
| **3D-pSOFA score** | Sham-operated | 0 *(0-0)* | 0 *(0-0.5)* | 0 *(0-1)* | 0 *(0-1)* | 0 *(0-0.5)* |
|  | **Non-septic** | 1 *(0-1.5)* | 1 *(0-2)* | 1 *(0-1)* | 1 *(0.5-2)* | 1 *(0-1)* |
| **5D-pSOFA score** | Sham-operated | 0 (0-0) | 0 *(0-0.5)* | 1 *(0-1)* | 1 *(0-1)* | 0 *(0-1)* |
|  | **Non-septic** | **1 *(0.5-1.5)*^x^** | **2 *(0.5-2)*^x^** | 1 *(0.5-1.5)* | **2 *(1-2)*^x^** | 1 *(0.5-2)* |

^X^*P*<0.05 vs sham-operated group; **P*<0.05 vs t=16 h

Supplemental digital content – Table 1B Changes in the hemodynamics in the sham-operated (n=9) and non-septic animals (n=5) during 16 h and 24 h of the monitoring period.

|  |  | Median *(25-75% percentiles)* | | | | |
| --- | --- | --- | --- | --- | --- | --- |
| **Parameters** | **Group** | **16 h** | **18 h** | **20 h** | **22 h** | **24 h** |
| **Heart rate** | Sham-operated | 60 *(51-65)* | 62 *(50-72)* | 61 *(57-67)* | 60 *(49-75)* | 60 *(56-70)* |
| (beat min ^-1^) | **Non-septic** | **97 *(65-103)*^x^** | 71 *(57-111)* | 102 *(55-122)* | **97 *(72-119)*^x^** | **104 *(70-130)*^x^** |
| **Cardiac index** | Sham-operated | 2.6 *(2.4-2.8)* | 2.4 *(2.4-3)* | 3 *(2.4-3.4)* | 2.8 *(2.4-3.1)* | 2.6 *(2.2-2.9)* |
| (L min^-1^ m^-2^) | **Non-septic** | 2.6 *(2.2-2.8)* | 2.8 *(2.4-3.8)* | 3.5 *(2.4-3.8)* | **3.4 *(3.3-3.6)*^x^** | **3.3 *(2.7-4.4)*^x^** |
| **SVI** | Sham-operated | 45 *(37-49)* | 42 *(39-46)* | 42 *(41-49)* | 44 *(35-46)* | 42 *(38-45)* |
| (ml beat^-1^ m^-2^) | **Non-septic** | **40 *(31-41)*^x^** | 42 *(32-43)* | **36 *(30-41)*^x^** | 33 *(29-45)* | 33 *(30-41)* |
| **SVRI** | Sham-operated | 3055 *(2554-3378)* | 3031 *(2448-3345)* | 2578 (2354-3692) | 2989 (2419-3977) | 3272 (2652-4120) |
| (dynes sec cm^-5^ m^-2^) | **Non-septic** | 2997 *(2347-3268)* | 2816 *(1994-3040)* | 2068 *(1785-3199)* | **2090 *(1939-2371)*^x^** | **2062 *(1720-3104)*^x^** |

^X^*P*<0.05 vs sham-operated group; **P*<0.05 vs t=16 h; SVI: stroke volume index; SVRI: systemic vascular resistance index

Supplemental digital content – Table 1C Changes in the oxygen dynamics, microcirculation and white blood cell count in the sham-operated (n=9) and non-septic animals (n=5) during the monitoring period.

|  |  | Median *(25-75% percentiles)* | | | | | |
| --- | --- | --- | --- | --- | --- | --- | --- |
| **Parameters** | **Group** | **0 h** | **16 h** | **18 h** | **20 h** | **22 h** | **24 h** |
| **DO_2_** | Sham-operated | **-** | 3409  *(3245-3836)* | 3142  *(3010-4300)* | 3831  *(3334-4139)* | 3544  *(2929-4173)* | 3375  *(2839-3769)* |
| (mL min^-1^) | **Non-septic** | **-** | 3643  *(3208-4309)* | 3888  *(3061-4551)* | 4567  *(2881-4694)* | **4432**  ***(3987-4894)*^x^** | *4005*  *(3212-5127)* |
| **VO_2_** | Sham-operated | **-** | 925 (372-982) | 710 (497-923) | 693 (560-1064) | 787 (757-955) | 898 (847-984) |
| (mL min^-1^) | **Non-septic** | **-** | 978 *(653-1125)* | **1038 *(799-1722)*^x^** | 986 *(889-1428)* | 1010 *(799-1714)* | **1127 *(928-1415)*^x^** |
| **Ex O_2_** | Sham-operated | **-** | 14 *(8-22)* | 11 *(9-24)* | 16 *(12-22)* | 13 *(11-29)* | 18 *(14-22)* |
| (%) | **Non-septic** | **-** | 24 *(16-35)* | **33 *(21-42)*^x^** | **32 *(20-38)*^x^** | 27 *(17-35)* | 27 *(24-33)* |
| **PPV** | Sham-operated | 98 *(93-100)* | 98 *(92-100)* | - | 92 *(85-100)* | - | 96 *(94-98)* |
| (%) | **Non-septic** | 95 *(92-98)* | 82 *(72-88)* | - | 95 *(86-97)* | - | 96 *(91-97)* |
| **White blood cell count** | Sham-operated | 10.7 *(9.2-13.2)* | 12.7 *(7.9-17.6)* | 12.5 *(9.3-13.4)* | 8.9 *(6.9-11.3)* | 8.3 *(6.9-11.2)* | 8.6 *(6.9-12.7)* |
| (x 10^9^ L^-1^) | **Non-septic** | 13.7 *(12.6-14.5)* | 7.6 *(5.8-11.6)* | 7.9 *(5.7-9.9)* | 7.6 *(5.1-10.9)* | 8.2 *(3.1-11.4)* | 8.5 *(3-12.1)* |

^X^*P*<0.05 vs sham-operated group; ~~*~~*~~P~~*~~<0.05 vs t=16 h;~~ DO_2_: oxygen delivery; VO_2_: oxygen consumption; Ex O_2_: oxygen extraction

Supplemental digital content – Table 1D Changes in markers of organ dysfunction in the sham-operated (n=9) and non-septic animals (n=5) during the monitoring period.

|  |  | Median *(25-75% percentiles)* | | | | | | |
| --- | --- | --- | --- | --- | --- | --- | --- | --- |
| **Parameters** | **Group** | **0 h** | **6 h** | **16 h** | **18 h** | **20 h** | **22 h** | **24 h** |
| **Bilirubin** | Sham-operated | 0.6 *(0.4-1)* | 0.6 *(0.5-1.5)* | 1.1 *(0.7-2)* | **1.3 *(1-2.2)**** | 0.6 *(0.5-1.2)* | 0.7 *(0.3-1.1)* | 0.9 *(0.5-1.3)* |
| (mmol L^-1^) | **Non-septic** | 0.5 *(0.3-0.7)* | 0.8 *(0.3-0.9)* | **1.4 *(1.1-3.6)**** | 1.1 *(0.6-1.4)* | 0.8 *(0.5-1.3)* | 0.8 *(0.6-1)* | 0.5 *(0.3-1.1)* |
| **Creatinine** | Sham-operated | 84 *(62-91)* | 82 *(70-100)* | 85 *(69-91)* | 86 *(62-97)* | 74 *(60-87)* | 79 *(57-85)* | 73 *(56-83)* |
| (mmol L^-1^) | **Non-septic** | 68 *(56-83)* | 65 *(49-82)* | **61 *(49-71)*^x^** | 69 *(51-75)* | 61 *(51-74)* | 65 *(54-67)* | 62 *(52-67)* |
| **De Ritis ratio** | Sham-operated | 0.5 *(0.4-0.6)* | 0.9 *(0.6-1.1)* | 0.9 *(0.6-1.7)* | 0.8 *(0.6-1.6)* | 0.8 *(0.5-1.5)* | 0.7 *(0.5-1.4)* | 0.7 *(0.5-1.6)* |
| (AST/ALT) | **Non-septic** | 0.5 *(0.3-0.7)* | 0.6 *(0.5-1)* | 0.6 *(0.6-1)* | 0.6 *(0.5-1.3)* | 0.5 *(0.5-1.1)* | 0.7 *(0.5-1)* | 0.6 *(0.5-0.9)* |
| **Albumin** | Sham-operated | 45 *(44-49)* | 45 *(42-50)* | 45 *(40-49)* | 42 *(39-46)* | 39 *(33-43)* | 37 *(35-43)* | **35 *(33-43)**** |
| (g L-^1^) | **Non-septic** | 43 *(39-48)* | 41 *(38-48)* | 39 *(36-43)* | **34 *(30-40)*^x^** | **30 *(30-35)*^x^*** | **30 *(26-36)**** | **30 *(24-31)*^x^*** |
| **Lactate** | Sham-operated | 1.4 *(1.1-1.5)* | 1.2 *(0.6-2.5)* | 1 *(0.8-1.2)* | 1 *(0.9-1.2)* | 0.7 *(0.6-0.9)* | 0.9 *(0.7-1.2)* | 0.8 *(0.6-1)* |
| (mmol L^-1^) | **Non-septic** | 1.5 *(1.3-2.4)* | 1.5 *(1.1-1.9)* | **1.7 *(1.1-1.9)*^x^** | **1.5 *(1-2)*^x^** | **1.5 *(1-1.7)*^x^** | **1.3 *(1-1.4)*^x^** | **1.7 *(1-2.5)*^x^** |

^X^*P*<0.05 vs sham-operated group; **P*<0.05 vs t=16 h
