# Supplementary material for: A Porcine Sepsis Model With Numerical Scoring for Early Prediction of Severity
Source: Front Med (Lausanne). 2022 May 9;9:867796. doi: 10.3389/fmed.2022.867796 (PMC9125192; doi:10.3389/fmed.2022.867796)
Supplement: Supplementary Table 2 — Changes in the parameters of terminated animals (with fulminant septic reaction) at t = 0 and 6 h. [file Table_2.DOCX]

Supplemental digital content – Table 2. Changes in the parameters of terminated animals (with fulminant septic reaction) at t=0 and 6 h.

|  | Median *(25-75% percentiles)* | |
| --- | --- | --- |
| **Venous blood** | **0 h** | **6 h** |
| **Lactate** (µmol L^-1^) | 1.6 *(1-1.8)* | 5.5 *(3.7-8.1)* |
| **O_2_ saturation** (%) | 84 *(74-85)* | 57 *(52-63)* |
| **Base excess** (mEq L^-1^) | 5.48 *(5.44-7.62)* | 0.22 *(-0.49-2.91)* |
| **Hemoglobin** (g dL^-1^) | 11.8 *(10-12.5)* | 16 *(12.9-17.2)* |
| **Bilirubin** (mmol L^-1^) | 1 *(0.5-1)* | 2.7 *(0.6-6.5)* |
| **Albumin** (mmol L^-1^) | 47 *(41-49)* | 39 *(32-45)* |
| **Creatinin** (mmol L^-1^) | 81 *(49-105)* | 89 *(66-113)* |
| **De Ritis ratio** (AST/ALT) | 0.3 (0.3-0.4) | 0.6 (0.5-0.7) |
