# Supplementary material for: A Porcine Sepsis Model With Numerical Scoring for Early Prediction of Severity
Source: Front Med (Lausanne). 2022 May 9;9:867796. doi: 10.3389/fmed.2022.867796 (PMC9125192; doi:10.3389/fmed.2022.867796)
Supplement: Supplementary Table 3 — Microbial composition of the sepsis-inducing inoculum expressed as the incidence of the current strain per total number of samples (%). [file Table_3.DOCX]

Supplemental digital content – Table 3. Microbial composition of the sepsis-inducing inoculum expressed as the incidence of the current strain per total number of samples (%)

| Microbial composition of feces (19 samples) | | | | | |
| --- | --- | --- | --- | --- | --- |
| Species | | | Number of samples containing the microorganism | Percentage of occurrence in the inoculum |  |
| Bacteria | Gram-negative | *Escherichia coli* | *19* | *100%* |  |
|  |  | *Klebsiella pneumoniae* | *7* | *37%* |  |
|  |  | *Pseudomonas aeruginosa* | *2* | *11%* |  |
|  |  | *Desulfovibrio piger* | *2* | *11%* |  |
|  |  | *Klebsiella oxytoca* | *2* | *11%* |  |
|  |  | *Prevotella sp.* | *1* | *5%* |  |
|  |  | *Pseudomonas stutzeri* | *1* | *5%* |  |
|  |  | *Proteus haueri* | *1* | *5%* |  |
|  | Gram-positive | *Enterococcus faecalis* | *4* | *21%* |  |
|  |  | *Enterococcus faecium* | *3* | *16%* |  |
|  |  | *Lactobacillus amylovorus* | *3* | *16%* |  |
|  |  | *Streptococcus suis* | *3* | *16%* |  |
|  |  | *Streptococcus orisratti* | *2* | *11%* |  |
|  |  | *Lactobacillus delbrueckii* | *2* | *11%* |  |
|  |  | *Streptococcus alactolyticus* | *2* | *11%* |  |
|  |  | *Lactobacillus acidophilus* | *1* | *5%* |  |
|  |  | *Bifidobacterium thermophilum* | *1* | *5%* |  |
|  |  | *Clostridium disporicum* | *1* | *5%* |  |
|  |  | *Streptococcus caballi* | *1* | *5%* |  |
|  |  | *Lactococcus garvieae* | *1* | *5%* |  |
|  |  | *Bacillus cereus* | *1* | *5%* |  |
|  |  | *Pediococcus pentosaceus* | *1* | *5%* |  |
|  |  | *Megasphera elsdenii* | *1* | *5%* |  |
|  |  | *Staphylococcus carnosus* | *1* | *5%* |  |
| Fungi | | *Rhodotorula mucilaginosa* | *6* | *32%* |  |
|  |  | *Candida lusitaniae* | *3* | *16%* |  |
|  |  | *Candida parapsilosis* | *3* | *16%* |  |
|  |  | *Candida glabrata* | *3* | *16%* |  |
|  |  | *Candida colliculosa* | *2* | *11%* |  |
|  |  | *Candida lambica* | *1* | *5%* |  |
